# Institutional Needs

**Published:** 1915-04-24

**Authors:** 


### Institutional Needs.

### THE SURGICAL MANUFACTURING COMPANY'S CATALOGUE.

In considering the interesting details contained in the catalogue issued by the Surgical Manufacturing Company, it is as well for London institutions to remember that the firm's factory is also situated within the Metropolis, and the short distance between it and the showrooms at 85 Mortimer Street enables prompt delivery to be secured, which is sometimes an important matter. There is not space here to review the catalogue in detail, but readers may be usefully reminded that special lists are supplied with detailed reference to the following classes of equipment: Surgical instruments, ambulance and emergency outfits, drums of sterilised dressings, dispensing sundries, and invalid furniture. A large variety of operating tables, including portable types, sterilisers, both steam and electric, instrument cabinets and instruments, aseptic ward lockers, both of enamelled steel and wood, ward screens, theatre and street ambulances, hospital sinks and lavatory fittings, food waggons, hospital beds and cots, wool and hair mattresses, couches for out-patients' consulting rooms, ward cabinets, and nurses' bedroom furniture are illustrated in great variety, and at prices whose figures may be judged by the fact that the Surgical Manufacturing Company make their own goods, and thus are independent of the middle profit.

